# Design and protocol for the Focusing on Clozapine Unresponsive Symptoms (FOCUS) trial: a randomised controlled trial

**DOI:** 10.1186/s12888-016-0983-6

**Published:** 2016-08-05

**Authors:** Melissa Pyle, John Norrie, Matthias Schwannauer, David Kingdon, Andrew Gumley, Douglas Turkington, Rory Byrne, Suzy Syrett, Graeme MacLennan, Robert Dudley, Hamish J. McLeod, Helen Griffiths, Samantha Bowe, Thomas R. E. Barnes, Paul French, Paul Hutton, Linda Davies, Anthony P. Morrison

**Affiliations:** 1 The Psychosis Research Unit, Department of Psychology, Greater Manchester West Mental Health NHS Foundation Trust, Prestwich, M25 3BL UK; 2 Department of Psychology, University of Manchester, Zochonis Building, Manchester, M13 9PL UK; 3 Centre for Healthcare Randomised Trials, Health Services Research Unit, University of Aberdeen, 3rd Floor Health Sciences Building, Aberdeen, AB25 2ZD UK; 4 Department of Clinical Psychology, University of Edinburgh. Medical School, Teviot Place, Edinburgh, EHY8 9AG UK; 5 University Department of Psychiatry, University of Southampton, Academic Centre, College Keep 4 - 12 Terminus Terrace, Southampton, SO14 3DT UK; 6 Institute of Health and Wellbeing, University of Glasgow, Gartnavel Royal Hospital, 1055 Great Western Road, Glasgow, G12 0XH UK; 7 Academic Psychiatry, Northumberland, Tyne and Wear NHS Foundation Trust, Centre for Aging and Vitality, Newcastle General Hospital, Westgate Road, Newcastle upon Tyne, NE4 6BE UK; 8 School of Psychology, Newcastle University, 4th Floor, Ridley Building 1, Queen Victoria Road, Newcastle Upon Tyne, NE1 7RU UK; 9 Centre for Mental Health, Imperial College London, Charing Cross Campus, St Dunstans Road, London, W6 8RP UK; 10 Institute of Psychology, Health and Society, University of Liverpool, Waterhouse Building, Block B, 2nd Floor, Liverpool, L69 3BX UK

**Keywords:** Schizophrenia, Psychosis, Clozapine-resistant, Cognitive behavioural therapy, Randomised controlled trial

## Abstract

**Background:**

For around a third of people with a diagnosis of schizophrenia, the condition proves to respond poorly to treatment with many typical and atypical antipsychotics. This is commonly referred to as treatment-resistant schizophrenia. Clozapine is the only antipsychotic with convincing efficacy for people whose symptoms are considered treatment-resistant to antipsychotic medication. However, 30–40 % of such conditions will have an insufficient response to the drug. Cognitive behavioural therapy has been shown to be an effective treatment for schizophrenia when delivered in combination with antipsychotic medication, with several meta-analyses showing robust support for this approach. However, the evidence for the effectiveness of cognitive behavioural therapy for people with a schizophrenia diagnosis whose symptoms are treatment-resistant to antipsychotic medication is limited. There is a clinical and economic need to evaluate treatments to improve outcomes for people with such conditions.

**Methods/design:**

A parallel group, prospective randomised, open, blinded evaluation of outcomes design will be used to compare a standardised cognitive behavioural therapy intervention added to treatment as usual versus treatment as usual alone (the comparator group) for individuals with a diagnosis of schizophrenia for whom an adequate trial of clozapine has either not been possible due to tolerability problems or was not associated with a sufficient therapeutic response. The trial will be conducted across five sites in the United Kingdom.

**Discussion:**

The recruitment target of 485 was achieved, with a final recruitment total of 487. This trial is the largest definitive, pragmatic clinical and cost-effectiveness trial of cognitive behavioural therapy for people with schizophrenia whose symptoms have failed to show an adequate response to clozapine treatment. Using a prognostic risk model, baseline information will be used to explore whether there are identifiable subgroups for which the treatment effect is greatest.

**Trial registration:**

Current Controlled Trials ISRCTN99672552. Registered 29^th^ November 2012.

## Background

In around a third of people with a diagnosis of schizophrenia, symptoms will respond poorly to standard treatment with antipsychotic medication. In a relatively small proportion of people, schizophrenia proves to be treatment-resistant in its early stages, failing to remit after the first exposure to antipsychotic medication [1]. However, more commonly treatment-resistance emerges over time: the symptoms become progressively less responsive to medication with subsequent relapses [2].

The clinical and economic costs of providing treatment for people who have a diagnosis of schizophrenia and whose symptoms have shown a poor response to treatment with antipsychotic medication is a major challenge in everyday psychiatry. It is estimated that the total societal cost of schizophrenia in the UK is £11.8 billion per year with the cost to the public sector estimated at £7.2 billion [3].

For people meeting diagnostic criteria for treatment-resistant schizophrenia, the economic costs are greater as longer-term residential and intensive community treatments are usually required [4, 5]. Even for a first episode of psychosis, symptoms may not remit in around 10 % over the first 12 months following initiation of treatment. There is clinical and economic need to evaluate treatments to improve outcomes for people who meet criteria for a diagnosis of schizophrenia with treatment-resistant symptoms.

Clozapine is the only antipsychotic with convincing evidence of efficacy for strictly-defined, treatment-resistant schizophrenia [2]. Meta-analyses have demonstrated that clozapine is superior to typical antipsychotics in improving positive and negative symptoms of schizophrenia [6]. However, clozapine has limited efficacy for such conditions, with 30–40 % showing an inadequate response to the drug [6]. In some people, a range of potentially serious side effects from clozapine, such as seizures, sedation and tachycardia, may prevent the optimal dose being reached. In the short term, metabolic side effects add to the known cardiometabolic risk factors in people diagnosed with a psychotic disorder and further increase the likelihood of developing diabetes and cardiovascular disease. The long-term adverse effects of antipsychotics are becoming increasingly recognised as problematic [7], so a demonstration of the benefits of alternative treatment options for this group of people is required. In recent years, the cognitive approach, successful in helping depression and anxiety, [8] has been applied to our understanding and treatment of psychosis. The basis of the cognitive approach is that the way that we interpret events will have consequences for how we feel and behave, and that such interpretations are often maintained by unhelpful thinking biases and behavioural responses. There have been several cognitive models of psychosis and psychotic experiences outlined [9–15], which suggest that it is how people interpret psychotic phenomena that accounts for distress and disability, rather than the psychotic experiences themselves. There are several comprehensive treatment manuals that describe the application of such models in greater detail [12–15]. Cognitive behavioural therapy for psychosis (CBTp) is effective and safe when delivered in combination with antipsychotic medication, with several meta-analyses showing robust support for this approach [16–20]. Recent meta-analyses have shown CBTp is more effective than other psychological therapies in reducing positive symptoms of psychosis, such as hallucinations and delusional beliefs [21].

To date, six randomised controlled trials (RCT) of CBT for people with a diagnosis of schizophrenia whose symptoms are treatment resistant to antipsychotic medication have been conducted [22–27] with a total sample of 361 participants. Of these, five trials targeted a participant population that met criteria similar to those for the initiation of clozapine treatment and only one small study has examined the efficacy of CBT for strictly defined clozapine-resistant psychosis [22]. Analysis of effect sizes on positive symptoms from the six existing trials that have focused on a treatment-resistant and/or clozapine-resistant population shows a mean effect size of 0.53. This is similar to the overall effect size of 0.4 from Wykes et al’s review of CBTp [17]. However, these effect sizes may be inflated as all studies were small and therefore the aggregate effect size may be biased upwards as a result of publication bias and poor quality of the methodology.

There are few studies from which to estimate the likely effect sizes of CBTp for people with a schizophrenia diagnosis who are unable to tolerate clozapine. However, results of a feasibility RCT of CBTp for people with schizophrenia spectrum disorders not taking antipsychotic medication found that CBTp significantly reduced psychiatric symptoms in comparison to treatment as usual and demonstrated it was both safe and acceptable [28].

CBTp has the potential to help alleviate symptoms and improve quality of life in people meeting criteria for treatment-resistant schizophrenia and there are suggestions that it can help those who are not taking antipsychotics. However, there is insufficient research regarding CBTp for people with an inadequate response to clozapine or who are unable to tolerate the drug. The FOCUS trial aims to address the question of whether, Cognitive Behavioural Therapy (CBT) is clinically and cost effective and an acceptable treatment for people with confirmed treatment-resistant schizophrenia that is poorly responsive to an adequate trial of clozapine monotherapy (or unable to tolerate such a trial)? We will test the hypotheses that:

1. In people with a diagnosis of schizophrenia spectrum disorder who have an inadequate response to or are unable to tolerate clozapine, CBTp plus Treatment As Usual (TAU) will lead to improvement in psychotic symptoms, measured using a psychiatric interview (PANSS), over a 21-month follow-up period compared with TAU alone.
2. CBTp plus TAU will lead to improved quality of life and user-defined recovery compared to TAU alone.
3. CBTp plus TAU will lead to a reduction in affective symptoms and negative symptoms compared to TAU alone.
4. CBTp plus TAU will be cost effective compared to TAU alone.

In addition, we will develop a prognostic risk model that identifies baseline factors that predict good response to CBTp.

## Methods and design

FOCUS is a parallel group, prospective, randomised, open, blinded evaluation (PROBE) of outcomes design comparing a standardised cognitive behavioural therapy for psychosis (CBTp) intervention added to treatment as usual (TAU) versus TAU alone (the comparator group) in individuals who are unable to tolerate clozapine or whose symptoms have failed to respond adequately to the drug. The trial has five sites across the United Kingdom (UK; Edinburgh, Glasgow, Manchester, Newcastle and Southampton). Our trial is designed as definitive, pragmatic, clinical and cost effectiveness trial. The study is funded by the National Institute for Health Research (NIHR) Heath Technology Assessment (HTA) program. The funders commissioned a call to answer the research question: what is the effectiveness and cost effectiveness of cognitive behaviour therapy for individuals who have treatment-resistant schizophrenia? The funder specified that important outcomes should be change in psychiatric symptoms and that other outcomes should be change in psychological measures, measures of function, frequency of relapse, health service use, quality of life, cost effectiveness. In addition, it was specified that the minimum follow-up should be 12 months and that the comparator should be treatment as usual. Recruitment of participants commenced on 1^st^ January 2013 and ended on 31^st^ May 2015. The trial is currently ongoing and data collection will continue until March 2017.

It would have been unethical to restrict the therapeutic options of the clinical teams participating; therefore, we did not ask referrers to withhold any treatment from the ‘treatment as usual’ options. Our approach is, accordingly, to record the use of all other medication and psychological therapies, document details of dosage, and ensure the follow-up of all randomised participants, irrespective of the interventions that they subsequently received.

Randomisation was in the ratio 1:1 to the two groups and was stratified by centre. Randomisation (at the individual level) was independent and concealed, using randomised-permuted blocks of random size, which was administered via a study-specific web-based portal (OpenCDMS, a web-based system developed with the National Institute for Mental Health (NIHR) Clinical Research Network (CRN) - Mental Health). The allocation was made known to the trial manager (in order to monitor adherence to the randomisation algorithm), the trial administrator and trial therapists by email. The allocation was also made known to the participant by letter from the trial administrator. The research assistants (RA) are blinded to the allocation code in order to protect against bias. Blindness is maintained using a wide range of measures, such as separate offices for the therapists and research assistants (RA), protocols for answering telephones, message taking and secretarial support, separate diaries, pigeon holes and data-file security. The Independent Data Monitoring Committee (IDMC) and Trial Steering Committee (TSC) monitor un-blinding by each centre to implement corrective action if necessary. If a blind break occurs, where possible, a new RA who is blind to allocation is identified for subsequent follow-up assessments.

The trial has been approved by the National Research Ethics Committee (NRES Committee Northwest-Lancaster) (12/NW/0520). Ethical approval was granted on 13^th^ August 2012. The current version of ethics approved protocol is version six and is dated 30/04/2015. A copy of this can be found here: http://www.nets.nihr.ac.uk/projects/hta/1010102 . The FOCUS Trial is sponsored by Greater Manchester West Mental Health NHS Foundation Trust. Full contact information for the sponsor can be found here: https://www.gmw.nhs.uk/research.

### Trial oversight

Oversight of the trial is provided by an Independent Data Monitoring Committee (IDMC). The IDMC is composed of two clinicians working in the area of psychosis (one of whom is the IDMC chair), a service user-led researcher and a statistician, all of whom are independent of the research team and have no competing interests. The IDMC is the only body involved in the FOCUS Trial, which has access to the unblinded comparative data in order to consider any ethical or safety reasons why the trial should not continue. The IDMC has convened annually since the trial commenced. The outcome of each IDMC meeting is reported to the funder via the minutes of the meeting. A copy of the IDMC charter is retained by the Trial Manager in the site file. Additionally, oversight is provided by a Trial Steering Committee (TSC) which is composed of independent members, a representative for the sponsor, the Chief Investigator (CI) and the Trial Manager. The independent members include: three clinicians (one of whom is the Chair of the TSC), a service user and a statistician. The TSC have convened annually since the trial commenced and interim reports regarding recruitment and compliance to follow-up have been provided via email in between the annual meetings. It is the role of the TSC to review the progress of the trial in terms of recruitment, withdrawals, blind breaks, patient safety (including over view of all adverse events reported to the CI) and adherence to the protocol. The outcome of each meeting is reported to the funder via the meeting minutes.

The Chair of the IDMC and TSC, and the funder review and agree all proposed changes to the protocol prior to research ethics committee review and approval. The sponsor is responsible for auditing the conduct of the trial.

### Interventions

Cognitive Behavioural Therapy for psychosis (CBTp) is delivered by appropriately qualified psychological therapists (clinical psychologists, counselling psychologists or other mental health professionals with specialist training in CBT). CBTp is delivered on an individual basis over a period of 9 months and includes up to 30 treatment sessions on an approximately weekly basis over the 9-month treatment window. CBTp is based on a specific cognitive model [11], since there is good evidence that CBTp based on empirically validated models is far superior to more eclectic cognitive behavioural approaches [29]. CBTp is based on a manualised approach in which, the assessment allows the development of an individualised formulation based on the cognitive model. The specific interventions are dependent on the individual formulation, but the range of permissible interventions is described in our published manuals [12, 30]. The aims of CBTp are to reduce distress (particularly that associated with psychotic symptoms) and improve quality of life. It is a collaborative therapy that works with the problems and goals that are agreed between patient and therapist. Thus, treatment targets often include positive symptoms, but frequently also include social issues such as improving relationships or developing meaningful social roles and issues of comorbidity including anxiety and depression. If comorbidity includes problematic drug or alcohol use, this can also be prioritised. Fidelity to the treatment protocol is ensured by weekly supervision of the therapists and assessed by rating audio recordings of therapy sessions using the Cognitive Therapy Scale-Revised [31]. All therapists in participating centres received initial training, which consisted of a residential week that covered assessment and formulation based on a specific cognitive model [11], milestones and assumptions for the therapy process and change strategies. Therapy supervision is provided by means of weekly face to face or telephone meetings. All CBTp sessions are audio recorded with the patient’s consent (patients may be asked to listen to the tapes as part of their homework) and a sample of tapes are rated using the CTSR in order to monitor fidelity and assist supervision; this is done throughout the lifetime of the trial in order to provide quality assurance and ensure corrective action can be taken if required. Following each session, therapists complete a session record that monitors the content of the session in terms of agenda targets, homework tasks and change strategies used. Thus, fidelity can be used as a mediating variable in supporting analyses. Additionally, this data is used throughout the trial to monitor adherence and address any adherence difficulties with therapist training sessions. The session records are anonymised and stored electronically in a database that is only accessible to specific members of staff who have been granted the necessary privileges. We would consider discontinuing therapy at the participants request to do so.

The control condition is treatment as usual. We do not ask clinical teams to withhold any treatment. Our assessments at baseline, 9 months (end of treatment) and 21 months (12-month follow-up) identify any risks to self or others that require immediate action. In addition, participants in both groups also receive a crisis card providing emergency contact details. All participants have an allocated keyworker/care coordinator and receive follow-up from a multi-disciplinary team within secondary mental health services. There is a clear safety protocol to alert clinicians should suicidal or dangerous ideation emerge. All routine or additional treatments in both conditions are monitored using the Economic Patient Questionnaire [32].

### Sample size

As we intended to estimate treatment effects across a range of outcomes, including recovery, in addition to psychiatric symptoms, we powered the study to detect a generic effect size of 0.33. With 194 participants per group, using a t-test with a significance level of 0.05 we would have 90 % power to detect an effect size of 0.33. A target recruitment of 485 allowed for a dropout rate of 20 %.

### Referral and recruitment

We recruited participants to the trial through referrals from community mental health care and inpatient settings across five sites in the UK (Glasgow, Edinburgh, Manchester, Newcastle and Southampton). In total, this has included 15 National Health Service (NHS) Trusts across the UK. Relevant mental health services were informed about the study through leaflets, attendance at team meetings and workshops/talks delivered by the research team. We also accepted self-referrals through study leaflets and posters, which were displayed in the waiting rooms of relevant mental health services. Referrals from both clinicians and service users (self-referrals) were taken by the research assistants working on the trial. Basic contact details and information pertaining to inclusion/exclusion criteria were taken at the point of referral, for self-referrals we informed the potential participant that we would need to contact their care team for referral information and sought verbal consent to do so. Potential participants were sent a participant information sheet and given at least 24 h to consider their involvement. Research assistant met with potential participants to discuss the participant information sheet and obtain informed consent. Recruitment was supported by the Scottish Mental Health Research Network (SMHRN) and the Mental Health Research Network (MHRN).

### Inclusion criteria

Patients were required to meet the following criteria to be eligible for enrolment:

1. A criterion level of persistent symptom severity despite an adequate trial of clozapine in terms of dosage, duration and adherence [33]:
  Treatment of clozapine at a stable dose of 400 mg or more (unless limited by tolerability) for at least 12 weeks, or if currently augmented with a second antipsychotic that this has been given for at least 12 weeks, without remission of psychotic symptoms, or have discontinued clozapine due to adverse reactions (including agranulocytosis) or lack of efficacy in the past 24 months.
2. Presence of at least one psychotic symptom with severity ≥4 (for hallucinations/delusions) or ≥5 (for suspiciousness/grandiosity) on the Positive and Negative Syndrome Scale (PANSS) in addition to a PANSS total score of at least 58, which is equivalent to a clinical global impression (CGI) of being at least mildly ill [34].
3. Be in contact with mental health services and have a care coordinator.
4. Either meet ICD-10 criteria for schizophrenia, schizoaffective disorder or delusional disorder or meet entry criteria for an Early Intervention for Psychosis service (operationally defined using PANSS) in order to allow for diagnostic uncertainty in early phases of psychosis.
5. Aged at least 16 years’ old
6. Competent and willing to provide written, informed consent.

### Exclusion criteria

1. Primary diagnosis of alcohol/substance dependence, where this is clearly the cause of their psychotic symptoms.
2. Developmental disability.
3. Non-English speaking.
4. Current receipt (or within the last 12 months) of structured CBTp from a qualified psychological therapist in accordance with NICE guideline recommendations (as opposed to more generic psychosocial interventions).

### Informed consent

If a person expressed an interest in the trial, they were provided with a NREC approved Participant Information Sheet and given at least 24 h to consider their involvement. Those who expressed an interested in participation were asked to provide written informed consent prior to their inclusion. Each participant was provided with a copy of their signed consent form. The consent form was also copied to the participant’s General Practitioner and allocated mental health professional from their care team. The research team retain a copy of all the consent forms.

### Data collection and management

All of the information that is collected about participants is strictly confidential. All personally identifiable information, such as participant name, is stored separately to the research data. All research data is anonymised with a trial ID number. Participants are made aware that although their data is strictly confidential and not shared out with the research team, this confidentiality can be broken if the participants are deemed a risk to themselves or others.

All personal information is stored securely in paper format (in locked cabinets in locked University and NHS Offices) and electronically in a password protected database, which is only accessible to the research team. Research data is stored securely in paper format in locked cabinets in University and NHS Offices and electronically on a study-specific web-based portal (OpenCDMS; see above for full details). Data is entered into OpenCDMS by the research assistants. In order to ensure the accuracy of the data entered into OpenCDMS the main outcome measure entries are checked for every participant. This involves checking the scores in the paper file against the scored entered into the electronic database OpenCDMS. An error rate of no more than 2 % is acceptable. This is done once all possible assessments for each time point have been completed. If the error rate is higher than 2 % advice will be sought from the trial statistician and methodologist regarding further data checking. The baseline period is now complete and the error rate was less than 2 % at all sites.

The final trial dataset will be managed and held by our Clinical Trials Unit (CTU), The Centre for Healthcare Randomised Trials (CHaRT) and requests for access to the dataset will be made in the first instance to the CI and then the CTU.

### Assessment of eligibility and outcome measures

**Fig. 1:**
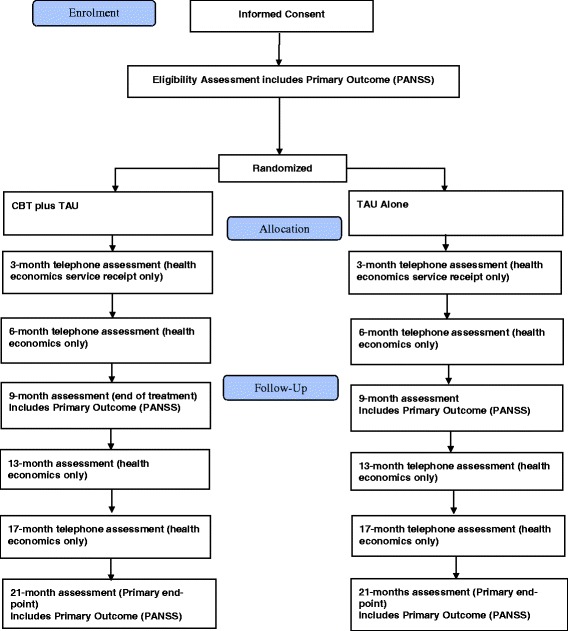
Trial flow diagram

The time schedule of enrolment, interventions and assessments for participants can be seen in Fig. 1. At the point of referral, basic information pertaining to the inclusion and exclusion criteria was sought from the clinical team, this included information relating to clozapine use, diagnosis, age, developmental disability, language and current/previous receipt of CBT. Research assistants (RA) met with potential participants before the first assessment to explain the trial, discuss the participant information sheet and provide an opportunity for the potential participant to ask questions. After this, the RA sought written informed consent from those who wish to take part in the trial. Eligibility on the PANSS was determined at the baseline assessment.

The primary outcome measure is the total PANSS score at 21-month assessment (12 months’ follow-up from the end of treatment). The primary outcome will allow for comparison with other published trials, inclusion of our results in any future systematic reviews and meta-analyses. Having the primary outcome at 12-month follow-up will allow an evaluation of the benefits of CBTp beyond the end of treatment. The PANSS is a 30-item rating scale designed to provide a comprehensive assessment of psychopathology in adult patients with schizophrenia. Five components have been reported: positive, negative, depression-anxiety, agitation-excitement, and disorganisation [35, 36]. The PANSS was completed at baseline assessment, 9-month follow-up and 21-month follow-up.

In addition to the PANSS, we administered a number of other measures at baseline assessment. The Psychotic Symptom Rating Scales (PSYRATS) [37] was used to assess dimensions of auditory hallucinations and delusional beliefs including frequency, preoccupation, location, loudness, conviction, and amount of unpleasant content, severity of unpleasant content, amount of distress, intensity of distress, degree and impairment of control. Personal and social occupational functioning was assessed by the Personal and Social Performance Scale (PSP) [38], this provided an assessment of functioning in four areas (socially useful activities, personal and social relationships, self-care and aggressive behaviours). Depression was assessed using the Calgary Depression Rating Scale for Schizophrenia (CDSS) [39]. Global illness severity was assessed using the Clinical Global Impression Scale (CGI) [40] and in addition to the clinician version a participant version (CGI-P) was utilised to obtain the participants perception of the severity of their mental health difficulties. Working memory was assessed using the Working Memory Letter Number (LN) span [41]. Furthermore, the participants were asked to complete a number of self-report questionnaires. These include: meta-worry subscale of the Anxious Thoughts Inventory (ATI [42]), The Process of Recovery Questionnaire (QPR [43]), the Alcohol Use Disorder Test (AUDIT [44]) and the Drug Abuse Screening Test (DAST [45]). Several self-report psychological measures were used to identify mechanisms of change and predictors of outcome these include the Interpretation of Voices Inventory, (IVI [46]) as a measure of appraisals of voices; the Beliefs About Paranoia Scale (BAPS [47]) as a measure of appraisals of paranoia; the Psychosis Attachment measure (PAM [48]) as a measure of attachment style; the Brief Core Schema Scale (BCSS [49]) as an measure of positive and negative beliefs about others; the Internalised Stigma Scale (ISMI [50]) and the Common Responses Questionnaire, which is a measure of common responses to psychotic experiences.

Following the baseline assessment, we invited all participants to attend two face-to-face assessments at 9 months (end of treatment) and 21 months (12-month follow-up). All of the measures detailed above are completed at the 9-month assessment with the addition of the Childhood Trauma Questionnaire (CTQ [51]), which is used to retrospectively assess histories of abuse and neglect. All of the measures listed above are also completed at 21-month follow-up with the exception of the Working Memory Letter Number (LN) and the CTQ. We also ask all participants at the 21-month follow-up to complete a self-report measure of potential adverse effects from trial involvement. This measure was developed specifically for use in the FOCUS trial to measure these broad categories: worsening difficulties; poor engagement (including low motivation); situational change; not getting benefit; stigma; increased conflict with others (care team, family etc.) and feeling better.

The Economic Patient Questionnaire (EPQ [32]) at baseline, 3, 6, 9, 13, 17 and 21 months is used to collect data from participants about the range and frequency of health and social care services used. The EPQ is used to identify hospital care and details of this are collected from case notes. The EPQ is administered by telephone at the 3, 6, 13 and 17 months’ follow-up. The Euroqol (EQ-5D) is a generic and validated health status questionnaire, shown to have acceptable validity in people with schizophrenia in European countries [52] and used in previous trials of interventions for people with schizophrenia [32, 53]. It was administered at baseline, 9 and 21 months’ follow-up. Quality adjusted life years (QALYs) gained from baseline to end of scheduled follow-up will be estimated as the number of weeks multiplied by the utility of observed survival. The utility values will be estimated from the Euroqol EQ-5D health status questionnaire completed at each follow-up assessment and the associated published societal utility tariffs.

Participants are offered choice regarding the duration of the of assessments visit, including the option of breaks and multiple assessment sessions. Assessment measures are clearly prioritised so that the most important are collected first to avoid missing data. We have a standard protocol for managing any distress that is associated with the completion of measures, which has been developed in collaboration with service users; this includes the offer of telephone contact within 48 h of assessments in order to check on participant well-being.

### Participant change of status

If a participant express that they are unsure whether they wish to remain in therapy, data collection or both, then they are provided with a range of options (reduced number of assessment measures, change in time and venue of their appointments, taking a break from therapy or delaying assessments) to ensure they have been provided with a flexible range of choices. If a participant wish to withdraw from therapy, data collection or both they can do so at any point without having to provide a reason. However, participants are offered the option of providing a reason for withdrawal if they wish to do so. Participants are also offered the choice to complete the self-report measure of potential adverse effects from the trial and return it to the trial manager in a pre-paid envelope.

### Safety monitoring and reporting

We record all events which meet National Research Ethics Committee (NREC) criteria for a serious adverse event (SAE), i.e. any untoward occurrence that: results in death, is life threatening, results in self-harm, results in harm to others, requires hospitalisation or prolongation hospitalisation, results in persistent or significant disability or incapacity, consists of a congenital anomaly or birth defect, or is otherwise considered medically significant by the investigator. In line with NREC guidance, a SAE occurring to a research participant is reported to the main REC where in the opinion of the Chief Investigator the event is related (i.e. resulted from any of the research procedures) and unexpected. To minimise the risk of bias in the appraisal of whether the event is related and unexpected, we seek an independent opinion for each event from a representative of the IDMC, who is an expert clinician in the area of psychosis. The IDMC and ITSC provide oversight of the adverse events and monitor for any patterns in events between the CBT plus TAU and TAU Alone arms of the trial.

In addition to the above, potential unwanted effects of trial participation will be reported as a PANSS 25 % or more deterioration, scores of six or more on the GCI-Improvement Scale and the adverse effects of trial participation self-report measure.

### Statistical analysis

Statistical analysis will take place after full recruitment and follow-up (i.e. there will be no interim analyses for efficacy, although an independent Data Monitoring Committee monitors trial progress and specifically any safety issues on a regular basis). The results of the trial will be presented following the standard CONSORT recommendations. Baseline and follow-up data will be summarised using the appropriate descriptive statistics and graphical summaries. Treatment effects will be presented with 95 % confidence intervals (CI). There will be no adjustment to secondary outcomes CIs for multiple testing. The primary outcome (PANSS total score at 21 months) will be analysed using a linear model that adjusts for pre-specified baseline covariates (baseline PANSS, sex, age) and includes a random effect for site. The development of treatment effects over time will be explored using a repeated measures mixed effects model that makes use of all available data, this assumes missing at random conditional on the observed covariates. The primary analysis will be intention-to-treat on available data (i.e. analyse as randomised). Secondary outcomes will be analysed in a similar way with generalised linear models appropriate for the distribution of the outcome.

We will try to identify subgroups for which the treatment effect is greatest using a prognostic risk model using baseline information. Response to treatment as measured by change in PANSS from baseline to 21 months will be explored in a general linear model. Covariates will include age; sex; baseline secondary outcome measures; childhood trauma; beliefs about self and others; working memory performance; and treatment allocated. Outcome analyses will be repeated excluding participants where the blind was broken to determine the robustness of the findings.

We will also address the influence of compliance and the therapeutic alliance between therapist and participant via causal or ‘mediation’ models. Traditional approaches to mediation [54] assume that confounding between the putative mediator and clinical outcome is absent (i.e. there is no omitted variable bias). We will compare the results of three sets of assumptions: (a) no confounding, (b) that we have measured and are able to adjust for all important confounders [55], and (c) that we are able to effectively adjust for unmeasured confounders (hidden confounding) using instrumental variable-based methods, specifically analyses based on principal stratification [56].

The sensitivities of all treatment effect estimates to missing outcome data will be explored; these models will explore the robustness of the treatment estimates to whatever small amount of missing data there is. The following strategy will be followed: firstly, the main analysis will use all available data that we believe are valid under the assumption of missing at random; secondly, a suite of sensitivity analysis to explore the robustness of the primary analysis to departures from assumptions that includes all randomised participants; finally, if required, sensitivity analyses will include multiple imputation, and imputing a range of values for missing data under missing not at random assumptions [57].

Data missing at baseline will be reported as such. If required for models for primary or secondary outcomes, continuous data will be imputed with the centre specific mean of that variable, missing binary/categorical data will include a missing indicator.

A copy of the intention to treat statistical analysis plan can be found here: http://w3.abdn.ac.uk/hsru/CHaRT/public/content/ShowPage.aspx?page=statistical-analysis-plans.

The economic analysis will estimate the costs of health and social care and quality adjusted life years (QALYs) from a broad societal perspective. This will include NHS secondary and primary care services, formal, independent and voluntary social care services and patients and family expenditure. The key determinants of total direct costs are expected to be those associated with the use of NHS hospital inpatient, outpatient and clinical services provided for the initial trial interventions and associated follow-up. These items comprised approximately 90 % of the total costs for participants in the recent CUtLASS trial who were randomised to second generation antipsychotics [32]. The time horizon for the economic analysis will be the 21 months from recruitment to end of scheduled follow-up. Quality adjusted life years will be the measure of health benefit from the primary analysis. Changes on key clinical measures from baseline to follow-up will be used in sensitivity analyses. QALYs will be measured using the EQ-5D health status measure and associated utility tariffs. Missing cost and QALY data will be treated as described above. Covariates for the cost effectiveness analyses will be identified from previous trials and descriptive analysis of the pooled baseline data.

Dissemination will occur via a number of methods which include publication of trial papers, conference presentations, book chapters, the Psychosis Research Unit website www.psychosisresearch.com and the HTA final report (monograph and trials directory). All authors must have contributed to one of each of the following: the design (conception and design or, acquisition of data and/or analysis and interpretation of data), the manuscript (drafting of the manuscript and/or critical revision of the manuscript for important intellectual content) and practical issues (statistical expertise, or obtaining funding, or administrative, technical, or material support, or supervision, or no additional contributions or other practical issues).

Participants will be informed of the results by being offered written and/or face-to-face feedback. This will be led by two co-applicants who are user-led researchers and members of the Psychosis Research Unit (PRU) Service User Research Group (SURG) have agreed to contribute to the process of communicating study findings, such as helping to generate a user-friendly sheet summarising the findings.

We intend to make data available to the scientific community with as few restrictions as feasible, while retaining exclusive use until the publication of major outputs has been completed.

## Discussion

For people diagnosed with schizophrenia who are unable to tolerate, or whose symptoms have a limited response to clozapine there is limited evidence for alternative treatments. There is therefore, an urgent need to evaluate evidence-based treatment options for this group of people. To our knowledge, our trial is the largest definitive trial of CBTp to date. It is the first multi-site, randomised, controlled trial of its size to investigate the clinical and cost effectiveness of CBTp in a strictly-defined, clozapine-resistant schizophrenia group. Recruitment to the trial has ended and we have recruited successfully to target. Indeed, we over-recruited to the trial by two participants, achieving a final total of 487 out of a target of 485.

Our trial has a number of unique features. A strength of the trial is that we collect the primary outcome data 12-months from the end of the treatment window. This provides an opportunity to determine the medium-range effects of CBTp and is unlike many other trials that have a primary outcome at end of treatment [58]. This is likely to be of particular importance given the findings from a meta-analysis of CBTp that the benefits of CBT may be delayed for up to a few months after treatment has ended [58].

Understanding and quantifying the risk of potential adverse effects of CBTp is important and early CBTp trials have been criticised for not giving this issue adequate consideration [59]. Klingberg et al. [60] have provided a useful template for assessing adverse effects as defined by the following criteria: (1) death by suicide, (2) suicide attempt, (3) suicidal crisis (explicit plan for serious suicidal activity without suicide attempt as defined by item 8, rating of 2 on the Calgary Depression Rating Scale for Schizophrenia; CDSS) and (4) severe symptomatic exacerbation (defined by the Clinical Global Impression Scale; CGI). We measure and report criteria 1–3 as part of National Research Ethics Committee requirements for recording and reporting serious adverse events. Additionally, we capture potential unwanted side effects from trial participation from scores on the CDSS and CGI. We will report scores of 6 or more on the GCI-Improvement Scale at 9 and 21 months’ follow-up. Furthermore, in order to capture the participants, own experiences and perspectives on trial participation we developed a bespoke measure of adverse effects from trial participation, which participants complete at the 21-month follow-up or are were offered at the point of withdrawal from the trial. Item generation was via a review of relevant literature on assessing adverse effects in psychotherapy trials [60, 61]. In relation to CBTp, we are also exploring the acceptability of CBTp through a qualitative evaluation of participants’ subjective experiences and perceptions of CBTp during the FOCUS trial. This aspect of the trial is led by service users.

It is unclear from the literature whether the treatment effects of CBTp may be greater for certain subgroups of people with a schizophrenia diagnosis. Such information would prove beneficial for people who have experience of psychosis and for their clinicians when making informed decisions about accessing CBTp. With this in mind, we seek to identify subgroups for which the treatment effect is greatest.

In summary, there an urgent need for evidence based treatments for people with a diagnosis of schizophrenia who are unable to tolerate or have experienced an inadequate response to clozapine. This trial addresses the question of whether CBTp is an effective treatment option for this group.

## Abbreviations

ATI, anxious thoughts inventory; AUDIT, alcohol use disorder identification test; BAPS, beliefs about paranoia scale; BCSS, brief core schema scale; CBT, cognitive behavioural therapy; CBTp, cognitive behavioural therapy for psychosis; CDSS, calgary depression rating scale for Schizophrenia; CGI, clinical global impression scale; CGI-P, clinical global impression-participant scale; CHaRT, centre for healthcare randomised trials; CI, confidence intervals; CRN, clinical research network; CTQ, childhood trauma questionnaire; DAST, drug abuse screening test; EPQ, economic patient questionnaire; FOCUS, focusing on clozapine unresponsive symptoms; IDMC, Independent Data Monitoring and Ethics Committee; ISMI, internalised stigma mental illness scale; ITSC, Independent Trial Steering Committee; IVI, interpretation of voices inventory; LN, letter number span; NICE, National Institute for Clinical Excellence; NIHR, National Institute for Health Research; NREC, National Research Ethics Committee; PAM, psychosis attachment measure; PANSS, positive and negative syndrome scale; PROBE, parallel group randomised outcome blinded evaluation; PSP, personal and social performance scale; PSYRATS, the psychotic symptoms rating scale; QPR, process of recovery questionnaire; RA, research assistant; RCT, randomised controlled trial; REC, Research Ethics Committee; SMHRN, Scottish Mental Health Research Network; TAU, treatment as usual; UK, United Kingdom
